# Using Raman Spectroscopy and Molecular Dynamics to Study Conformation Changes of Sodium Lauryl Ether Sulfate Molecules

**DOI:** 10.1021/acs.jpcb.3c02022

**Published:** 2023-05-16

**Authors:** Rachel L. Hendrikse, Andrew E. Bayly, Peter K. Jimack, Xiaojun Lai

**Affiliations:** † School of Chemical and Process Engineering, University of Leeds, Leeds, LS2 9JT, United Kingdom; ‡ EPSRC Centre for Doctoral Training in Fluid Dynamics at Leeds, University of Leeds, Leeds, LS2 9JT, United Kingdom

## Abstract

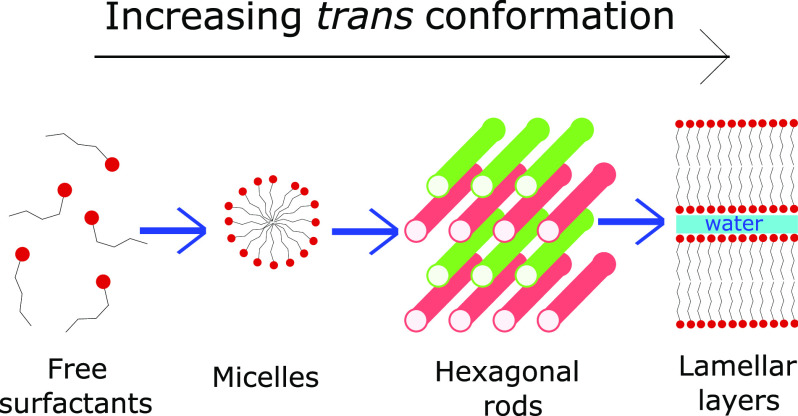

A study using both Raman spectroscopy and molecular dynamics (MD) simulations was carried out for alkyl ethoxysulfate (AES) surfactants at various concentrations in solution. Direct comparison between experiment and simulation shows that the conformational changes observed in MD are in good agreement with those obtained via Raman spectroscopy. We show that there is an increase in the relative number of *trans* conformations with increasing concentration and illustrate the relationship between phase structure and molecular conformation, which is often speculated but difficult to confirm. Our results open up the possibility of applying MD to other surfactants, with the aim of analyzing conformational behavior, which can typically be difficult to study experimentally using spectroscopy methods, due to large numbers of vibrational modes present in large complex molecules.

## 1 Introduction

Surfactant molecules will self-assemble into different phases in aqueous solutions at concentrations that are above the critical micelle concentration (CMC). Common structures include micellar solutions, hexagonal, bicontinuous cubic, and lamellar structures. Molecular conformation in surfactant solutions has been shown to be strongly dependent on concentration,^1,2^ largely due to the influence of self-assembled structure. A study of the conformational properties of different phases helps us to understand their structure more thoroughly at a molecular level. Phase boundaries are typically identified via a combination of experimental approaches, including polarized optical microscopy,^3−6^ small-angle X-ray scattering,^3−6^ rheology,^4^ differential calorimetric measurements,^7^ and/or NMR.^5,6^ However, the determined phase boundaries can differ between experimental methods. Phase identification can become difficult close to phase boundaries, which is sometimes due to the coexistence of different phases or phases that are poorly defined. Therefore, a study into conformational behavior may also aid the identification of the structure present when the results from different experimental approaches produce conflicting results.

In this work, we study alkyl ethoxysulfates (AES), which are common anionic surfactants where molecules can vary in their degree of ethoxylation *n* and alkyl chain length *x*. Specifically in this work, we study the behavior of AES solutions which possess a distribution in the number of ethylene oxide groups with a mean value of *n̅* ≈ 1.03, which typically undergoes micellar → hexagonal → lamellar phase transition^8^ with increasing concentration in water. This work uses Raman spectroscopy to quantify conformational changes that result from transition between phase structures for AES/water solutions, which could not be found in the existing literature. Additional rheological measurements are performed for micellar solutions at various concentrations. Despite the abundance of AES used in surfactant-containing products across the industry, the structure of micellar phases of AES solutions and the impact of this structure on the solution viscosity remain poorly understood. We aim to contribute to the understanding of the rheological behavior via an understanding of the conformational changes of the molecule.

While infrared spectroscopy^2,9,10^ (IR) is a common experimental tool for conformation studies, Raman spectral methods are particularly suited to studying surfactant solutions, since Raman is sensitive to the scattering of C–H bonds dominating the hydrophobic tail. Raman spectroscopy is also insensitive to O–H bonds (relative to IR methods), allowing us to study the behavior of the surfactant molecules with minimal obscuring from water vibrational modes. This is due to the fact that Raman spectral methods are sensitive to changes in the polarizability of a molecule, whereas IR spectroscopy depends on changes in the dipole moment. Therefore, the magnitude of scattered Raman intensity correlates with the polarizability of the molecule, meaning that polar bonds (e.g., C–O and O–H) are relatively weak Raman scatterers, while neutral bonds (e.g., C–C and C–H) have large changes in polarizability during a vibration. IR interacts very strongly with water molecules, with these vibrational modes being so large that they usually obscure other vibrational modes in the sample.

Conformational changes in this work are primarily quantified by analyzing the ratio of *trans* to *gauche* conformations in the hydrocarbon chain. Raman spectroscopy is particularly suited to studying the hydrocarbon chain and has been applied to surfactant and lipid systems by a variety of authors.^11−15^ In *n*-alkanes with up to 18 carbon atoms, the all-*trans* configuration has been shown to be the most stable.^16^ While the *gauche* conformation is less energetically favorable for hydrocarbon chains, they are present in solution because small amounts of energy are sufficient to overcome the energy barrier between *trans* and *gauche* conformations. It has been shown that the abundance of *gauche* and *trans* segments can abruptly change as a result of a phase transition,^2,13,17^ as the *trans* conformation becomes more energetically favorable at higher concentrations, where the energy barriers are harder to overcome. One drawback of Raman spectroscopy is that, while specific spectra peaks can be assigned to various conformations, the exact relationship between a peak’s magnitude and the abundance of said conformation in solution is unknown. Therefore, while Raman spectroscopy can be used to study relative changes, it is difficult to quantify the magnitude of any changes observed across the phase diagram.

A typical computational approach for studying conformational behavior is molecular dynamics (MD).^18,19^ However, the use of all-atom MD for surfactant systems is challenging because it is unable to reach the long time and length scales required for the self-assembly of micelles which possess aggregation numbers that are comparable to experiment. Beginning an all-atom simulation with a random initial configuration usually leads to significantly under-predicted micelle sizes.^20^ This means that existing studies have typically taken the approach to preassemble molecules into their desired arrangement prior to simulation,^19,21,22^ or generate their initial configurations through back-mapping coarse-grained simulations.^23^ While this reduces the simulation time required, it also raises the question of whether the prearranged configuration is truly an accurate representation of the equilibrium arrangement. For example, calculations performed on preassembled micelles rely upon the assumption that the micellar aggregation number is already accurately known. Therefore, in an ideal situation, one would want to begin a simulation from a random initial configuration of molecules in order to uncover true equilibrium structural behavior.

Due to the various pros and cons associated with prearranging micelles vs random initial configurations, we perform a variety of both types of all-atom simulations in this work. Simulation studies allow us to quantify the magnitude of the conformational changes more easily and also help us to understand the effect that phase structure has on the conformation in more detail. Consequently, in this work, we study the conformation of surfactant molecules using both experimental and numerical techniques with the aim of directly comparing the results from the two.

## 2 Materials and Methods

### 2.1 Materials

The AES paste used is a commercial grade of surfactant supplied by Procter & Gamble containing 70% AES surfactants. The surfactant paste was a commercial grade of paste with no subsequent additions or purification. As a result of the manufacturing process, a low level of impurities would be expected in addition to water and surfactant. AES surfactants have the chemical formula CH_3_(CH_2_)_*x*_(OCH_2_CH_2_)_*n*_OSO_3_Na where *x* varies between *x* = 11 and 15 (odd values of *x* only). Chains of length *x* = 11 dominate the distribution, such that the average is *x̅* ≈ 11.8. Similarly, the number of ethoxy groups *n* varies in the range *n* = 0–6 and takes an average value of *n̅* ≈ 1.03 (Table 1).

**Table 1:** Distribution of Ethoxy Groups *n* in AES Paste

***n***	**0**	**1**	**2**	**3**	**4**	**5**	**6**
(%)	49	24	13	7	4	2	1

Samples are prepared by mixing AES paste with deionized water to create the desired concentration and leaving the sample to stand at room temperature for an extended period. Solutions that went on to form micellar solutions were left to equilibrate for at least 2 weeks before measurement, and all other samples at higher concentrations were allowed to stand for at least 12 weeks. Additional rheological measurements are performed on sodium dodecyl sulfate (SDS) micellar solutions to allow for comparison (where SDS is essentially AES with *x* = 11 and *n* = 0). The SDS (99+%) was purchased from Sigma-Aldrich.

**Figure 1:**
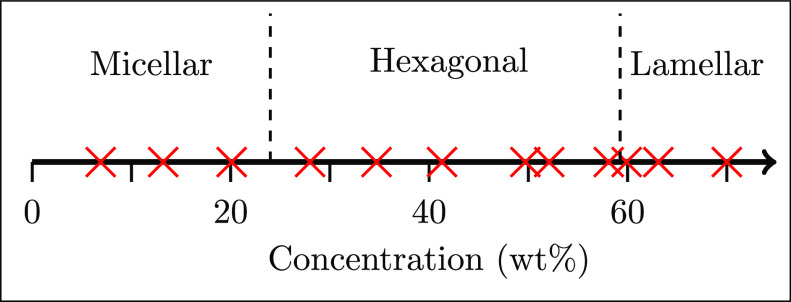
Summary of the phase diagram as presented in Hendrikse et al.,^8^ where symbols indicate concentrations sampled.

The phase transitions of AES were identified in a previous study^8^ (summarized in Figure 1) via polarized optical microscopy (POM) and rheological measurements, placing the micellar–hexagonal phase transition within the region of 20.1–28.0% and the hexagonal–lamellar transition in the region of 58.6–59.9%.

### 2.2 Experimental Methods

All experimental measurements were performed at 25 °C. Raman spectroscopy measurements are performed using the RamanRxn1 from Kaiser Optical Systems, which uses a wavelength of 785 nm. We perform 6 averaged 30 s acquisitions per sample.

Rheology measurements on the micellar phases were performed using an Anton Paar Physica MCR301 Rheometer. We perform measurements of solutions in the concentration range 7–20%, using a concentric cylinder geometry (27 mm diameter cylinder and gap size of 1 mm). Measurements are performed at a variety of shear rates, where the lower bound on the shear rate is dictated by the torque range of the rheometer and the upper bound is limited by the emergence of secondary flows (at which point Couette flow can not be assumed).

### 2.3 Molecular Dynamics

#### 2.3.1 Overview

Atomistic molecular dynamics simulations are conducted using the LAMMPS (Large-scale Atomic/Molecular Massively Parallel Simulator) code.^24^ Force field parameters are obtained from the Automated Topology Builder (ATB) and Repository.^25^ We perform molecular dynamics simulations for surfactants with monodisperse tail length *x* = 11, for two different degrees of ethoxylation: sodium dodecyl sulfate (SDS), corresponding to *n* = 0, and sodium laureth-1 sulfate (SLE1S), corresponding to *n* = 1. We chose to perform monodisperse simulations, as opposed to polydisperse in *x* and *n*, because it is difficult to simulate a distribution of sizes accurately due to the relatively small number of molecules that can be simulated. The *n* = 0 and *n* = 1 molecules are chosen for study since they contribute to the bulk of the AES size distribution. Simulations are performed in the micellar (*c* ≈ 20 wt %) and lamellar (*c* ≈ 70 wt %) regions of the phase diagram, excluding the middle range of concentrations. This exclusion is due to the difficulty generating hexagonal phases for study, which have even been shown to be challenging in coarse-grained studies.^8^

The TIP3P model^26^ with a long-range Coulomb solver^27^ is used for simulating water molecules. The choice of the TIP3P model is consistent with other researchers for studying micelle formation,^23,28^ although it has been shown that the choice of water model does not affect the predictions for the mean aggregation number of micelles.^23^ All simulations in this work are conducted in a cubic box with periodic boundary conditions.

A selection of simulations are performed with molecules initialized at randomly generated positions, where molecules are generated with an initial density which is lower than the target mass density. Other simulations are conducted with nonrandom placement, which will be discussed later in this section. The practice of initializing with a lower density than the known experimental density is found to aid in the equilibration of the system. Following the initial generation of positions, an energy minimization is conducted, iteratively adjusting atom coordinates to minimize the total potential energy of the system. This procedure creates the initial configuration for the system.

We simulate an isothermal–isobaric (NPT) ensemble using a Nosé–Hoover thermostat and barostat. The simulation is conducted at a constant pressure of 1 atm and a temperature of 300 K. Over the course of the simulation, the box volume decreased until the density reached a steady-state value. A time step of 1 fs was used throughout the simulations. The Lennard-Jones interactions were cut off at 1.5 nm, and the particle–particle particle–mesh (PPPM) Ewald method was used for the long-range electrostatic interactions.

#### 2.3.2 Set-up

Simulations of *n* = 0 molecules with random initial configurations are all performed at 20 wt %, where various box sizes are investigated. The different-sized simulations are defined by the total number of molecules inside the box, and four cases are run corresponding to 5,000, 10,000, 15,000, and 20,000 molecules. Table 2 provides the exact number of water and surfactant molecules in each case. Each simulation case is performed over a similar time frame, with the domain divided across the same number of processors (64 cores, i.e., 4 × 4 × 4 division). Therefore simulations containing fewer molecules run for a larger number of time steps and vice versa. The number of time steps achieved for the different simulation cases are as follows: 6.1 × 10^8^ (*N*_T_ = 5,000), 3.5 × 10^8^ (*N*_T_ = 10,000), 2.6 × 10^8^ (*N*_T_ = 15,000), and 2.0 × 10^8^ (*N*_T_ = 20,000). Note that the larger simulations are run for slightly longer than the smaller ones, in order to try and increase the run time for the cases with more atoms (giving the appearance of nonlinear scaling).

**Table 2:** Details of Simulations Performed for Initially Random Systems, Where *N*_T_ Is the Total Number of Molecules in the System and *N*_W_ and *N*_S_ Are the Number of Water and Surfactant Molecules, Respectively

*N*_T_	*N*_S_	*N*_W_
5,000	83	4917
10,000	167	9833
15,000	250	14750
20,000	334	19666

While these simulations are unable to reach aggregation numbers which are comparable with those obtained experimentally, due to computational limitations on the time scale involved, the dependence of quantities as related to their aggregation size *N* and box size can be studied. When the box size is smaller, the simulation has a better chance of reaching larger aggregation numbers that are comparable to experimental values. However, there will be very few (approximately one or two) micelles in the simulation box, making it difficult to study the properties of micelles in relation to *N*. The benefit of larger simulation boxes is that a number of micelles can be generated, allowing us to study properties as a function of size. However, the drawback, in this case, is that the micelles will likely be smaller and farther from the experimental aggregation number. Our choice of using various box sizes means that we can combine the benefits of the two approaches, allowing us to study as a function of *N* as well as achieving realistic micelle sizes.

Additional simulations are performed for preassembled micelles, lamellar phases and free monomers for both types (*n* = 0 and *n* = 1) of molecules. This allows us to investigate the impact of micelle formation and phase transition on conformation. These simulations are described in Table 3, where the number of molecules is chosen to produce the box sizes required for a particular phase. For the single molecules, this means ensuring that the box edge length is appropriately longer than the length of a single molecule. For SDS (*n* = 0), this is expected to be around 2 nm, while the addition of the ethylene oxide unit in SLE1S (*n* = 1) is expected to increase the length of the molecule by around 0.4 nm. For the individual micelles, the box size should be at least able to contain a single spherical micelle, estimated to be approximately twice the diameter of a single molecule. For lamellar phases, we chose the number of molecules to produce a single bilayer with the correct experimental *d*-spacing. For AES this is experimentally reported as 4.05 nm.^29^ SDS lamellar phases are not found at high concentrations experimentally^7,30^ and are usually reported as mixtures of crystalline SDS and various mesophases. Therefore, instead, we simulate to produce an edge length slightly smaller than that for SLE1S, since there is evidence of a reduced *d*-spacing with fewer degrees of ethoxylation.^8,31^

**Table 3:** Details of Simulations Performed for Pre-Assembled Systems Where *N*_W_ and *N*_S_ Are the Number of Water and Surfactant Molecules, Respectively^a^

Molecule	Phase	*N*_S_	*N*_W_	*c* (wt %)	*L* (nm)
*n* = 0	Monomer	1	1000	1.6	3.07
	Micellar	70	4480	20.0	5.37
	Micellar	100	8000	16.7	6.45
	Lamellar	98	500	75.8	3.75
*n* = 1	Monomer	1	1000	1.81	3.07
	Micellar	50	8000	10.3	6.31
	Micellar	100	8000	18.7	6.49
	Lamellar	93	786	68.6	3.99

a *L* is the final box edge length after density equilibration, and *c* is the concentration which results from these choices of molecules.

The initial configurations for the micellar and lamellar phase structures are generated to enable quick equilibration of the system. For the lamellar phases, this means preassembling a single micelle in the center of the simulation box. To generate the lamellar structures, we generate the water and surfactant molecules in two separate regions of the box, to allow the surfactant bilayer to generate without needing to separate out from the water molecules first (as it would in an initially random configuration).

## 3 Results and Discussion

### 3.1 Raman Spectroscopy

**Figure 2:**
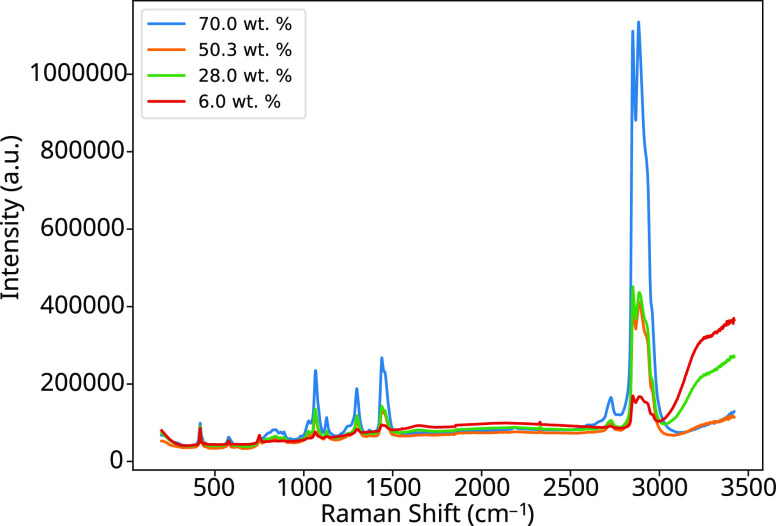
Raman spectra of aqueous AES solutions at concentrations of 6.0, 28.0, 50.3, and 70.0 wt %. Spectra are shown prior to background removal in the range 200–3425 cm^–1^.

**Figure 3:**
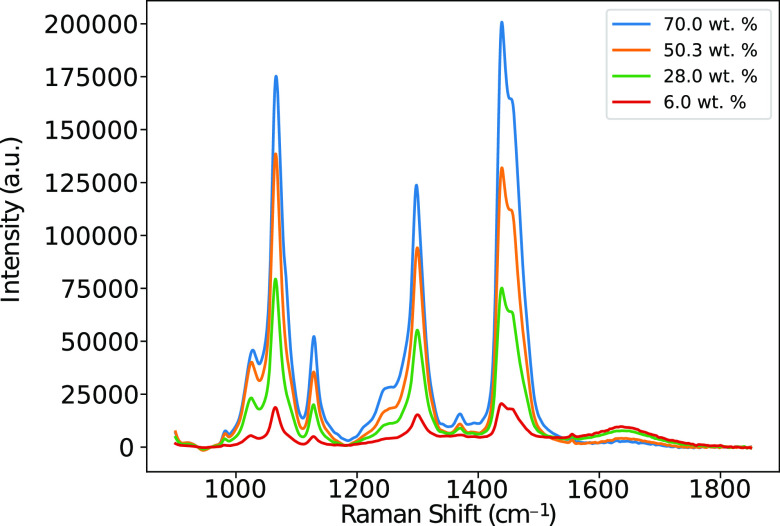
Raman spectra in the range 800–1800 cm^–1^ for aqueous AES solutions at concentrations of 6.0, 28.0, 50.3, and 70.0 wt %. Background removal has been applied.

**Figure 4:**
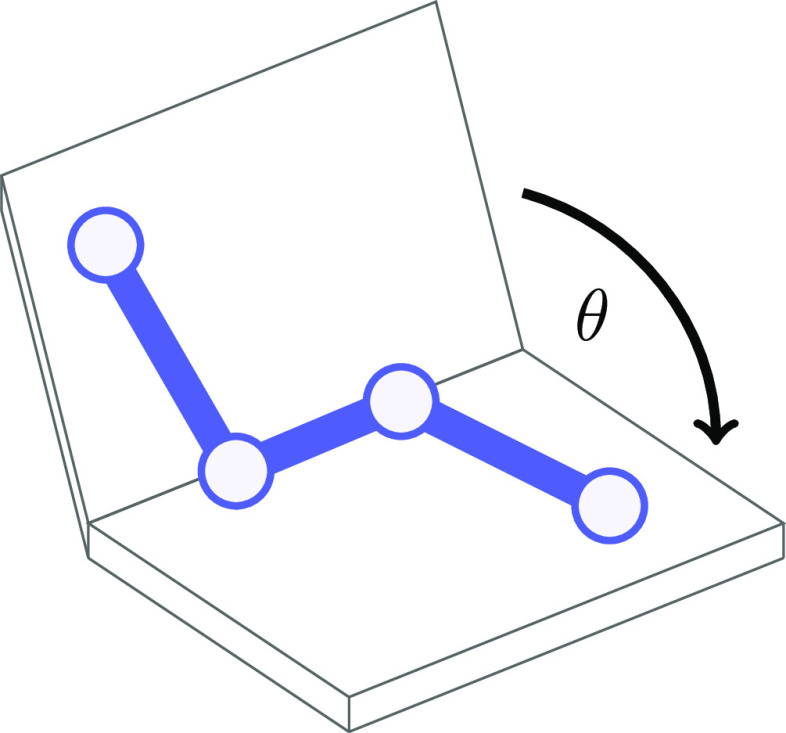
Illustration of the theoretical dihedral angle between atoms θ. Special cases of θ: θ = 180° (*trans*), θ = +60° (*gauche*^+^), and θ = −60° (*gauche*^–^).

Examples of Raman spectra for AES solutions consisting of different concentrations are shown in Figure 2, where equipment can measure in the range 200–3425 cm^–1^. The bands that appear with the largest intensity are from the C–H stretching modes between 2800 and 3000 cm^–1^ and from the O–H bonds of water at 3000–3800 cm^–1^. There are also a number of medium-magnitude peaks of interest in the region from 900 to 1800 cm^–1^. Other regions contain a number of peaks of smaller magnitude; however, these are not analyzed in this work due to the large amount of noise they contain. Figure 3 shows a reduced plot of the 800–1800 cm^–1^ region alone so that the peaks in this region can be more closely seen since the peaks at 2800–3000 cm^–1^ dominate the spectrum. Conformational changes are analyzed in this work by assigning particular peaks in the spectra to be dominated by vibrational modes originating from either *trans* or *gauche* conformations in the hydrocarbon chain, illustrated in Figure 4. The ratios of the intensity of peaks are used to quantify changes in the abundance of particular conformations, as a function of varying AES concentration.

#### 3.1.1 Peak Assignment

##### 3000–3800 cm^–1^

The O–H stretching band at 3000–3800 cm^–1^ is inhomogeneously broad and is usually fitted as a number of overlapping component sub-bands related to different O–H vibrational modes. For example, one possible fit places sub-bands at 3041, 3232, 3430, 3557, and 3635 cm^–1^.^32^ This band is in the proximity to the bands produced from CH_2_ and CH_3_ stretching in the 2800–3000 cm^–1^ range. Indeed, there can be considerable overlap between these two regions,^33^ so although we will focus primarily on changes produced in the vibrational modes from the surfactant molecules, the bands produced from the water molecules cannot be ignored, particularly at low concentrations. We find that the water vibrations in this region can be approximated using only a two-component fit, with maxima centered at around 3250 and 3460 cm^–1^.

##### 1000–1150 cm^–1^

In hydrocarbon chains, it is usually found that three Raman bands appear in the region of 1000–1150 cm^–1^.^17,34,35^ Two bands appear at around 1060 and 1130 cm^–1^, which can be assigned to the C–C stretching of *trans* segments. Another band appears at around 1080 cm^–1^ which is assigned to C–C stretching modes of *gauche* segments, and this region has been widely used for monitoring conformational changes.^11,17,34−36^ The ratio of these peaks can be used to evaluate the average number of *trans* bonds appearing in solution. These peaks can also undergo frequency shifts as a result of the phase change.^11^ In SDS molecules, some authors attribute part of the peak at around 1080 cm^–1^ to being the result of symmetrical stretching of SO_3_; Picquart^11^ considers this and argues that there is likely significant obscuring of the C–C stretching modes from SO_3_ stretching in this region.

A further added complication in this region is that it is highly likely that there are a number of hidden contributions from C–O bonds. For example, polyethylene glycol H(OCH_2_CH_2_)_*n*_OH shows Raman peaks in aqueous solution at around 1040, 1060, 1120, and 1140 cm^–1^, all corresponding to C–O–C modes.^37,38^ Similarly placed bands are reported in the spectra of dimethoxyethane.^39^ Despite this being the most common spectral range for analyzing the *trans*/*gauche* ratio in hydrocarbon chains, this is difficult for AES-like molecules due to the number of overlapping peaks expected in the region.

##### 1200–1400 cm^–1^

In the region 1200–1400 cm^–1^, the spectrum is dominated by a peak at 1295 cm^–1^, which primarily originates from CH_2_ twisting in the hydrocarbon chain.^40−42^ Other peaks in this region may include a vibration at around 1300 cm^–1^, related to a SO_4_ stretch,^41^ although this mode is weak in comparison.^10^ The CH_2_ twisting mode has been reported to be moderately sensitive to chain conformation, with band narrowing found upon increased conformational order.^43^ These observations are supported by quantum mechanical calculations, which show that consecutive *trans* bonds produce a narrow band, whereas conformations with a mixture of *trans* and *gauche* dihedral angles produce broader spectral responses.^44^ However, the overall integral of the peak at 1295 cm^–1^ is generally conformation independent,^45^ and thus can be used as a reference peak.

##### 1400–1600 cm^–1^

We find that the CH_2_ scissoring band presents two components: a main band at 1440 cm^–1^ and a shoulder at 1460 cm^–1^. The shoulder at around 1460 cm^–1^ is usually attributed to the CH_3_ mode.^46^ These modes have been shown to be sensitive to phase structural changes, having been shown to change with temperature and concentration, e.g., a decrease in frequency separation of the two components with increasing concentration.^43^ However, how these changes relate directly to the conformational shape is not well-known. Furthermore, there is most likely at least one peak of unknown origin in this band with curve decomposition requiring at least three modes to obtain a reasonable fit. Therefore, we will not analyze this region for evidence of conformational changes.

##### 2800–3000 cm^–1^

In the 2800–3000 cm^–1^ region, there is a broad band which is made up of multiple individual sub-band contributions, attributed to CH_3_ and CH_2_ symmetric and asymmetric stretching. Assigning peaks within this region has historically been controversial, with different authors assigning peaks as originating from different vibrational modes. It is generally agreed that a mode at around 2850 cm^–1^ originates from ν_s_(CH_2_) vibrations and the mode at 2960 cm^–1^ from ν_as_(CH_3_) vibrations.^15,47−56^ A peak also usually manifests at around 2880 cm^–1^, which across the literature has been assigned differently. One common assignment attributes this peak to ν_s_(CH_3_)^15,47−49^ vibrations, while others categorize it as having ν_as_(CH_2_) origin.^50−54^ DFT calculations^55,56^ support the assignment of ν_as_(CH_2_), and therefore, this is the choice we make in this work. We follow the same general peak assignment as Shemouratov et al.,^50^ assigning modes at around 2850 and 2880 cm^–1^ to the symmetric and antisymmetric stretching vibrations of the CH_2_ group in the *trans*-conformers. The *gauche*-conformers are assigned to frequencies at 2875 and 2925 cm^–1^, and peaks at 2940 and 2960 cm^–1^ are assigned to the symmetric and antisymmetric vibrations of CH_3_. A summary of peak assignment in the regions 1200–1400 and 2800–3000 cm^–1^ is shown in Table 4. These assignments originate from the previous work of theoretical,^53,57^ computational,^52,55,56^ and experimental^50^ approaches.

**Table 4:** Assignment of Peaks in the Raman Spectra of AES Solutions Where ν Denotes Stretching and τ Twisting

Wave number (cm^–1^)	Assignment
1295	τ(CH_2_)
2850	ν_s_(CH_2_) (*trans*)
2880	ν_as_(CH_2_) (*trans*)
2875	ν_s_(CH_2_) (*gauche*)
2925	ν_as_(CH_2_) (*gauche*)
2940	ν_s_(CH_3_)
2960	ν_as_(CH_3_)

#### 3.1.2 Analysis

**Figure 5:**
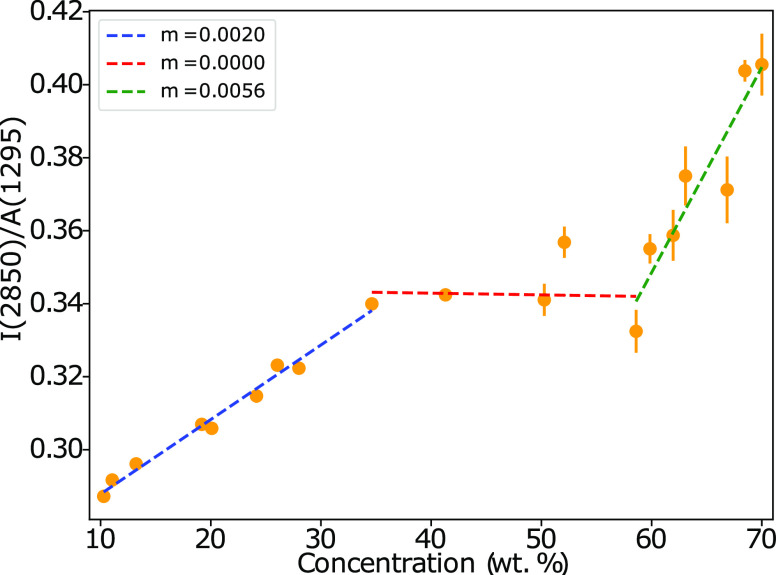
The ratio of the intensity at *I*(2850) to the area of peak at *A*(1295). Error bars are calculated as the standard error of multiple readings, and *m* is the gradient of linear fits within each subregion.

The ratio of peak intensity *I*(2850) to the integrated area of peak at *A*(1295) should increase as the number of *trans* conformations increases, based on assignments in Table 4. This can be considered to be peak scaling using the CH_2_ twisting mode, and this reference peak has also been chosen by other authors.^58^ The ratio *I*(2850)/*A*(1295) changes as a function of the AES concentration (Figure 5), indicating that increasing concentration leads to a greater ratio of *trans* conformations relative to *gauche*. Three distinct regions are identified and are broadly assigned as corresponding to the micellar, hexagonal, and lamellar phases (in order of increasing surfactant concentration). For each of the three subregions, a different relationship between conformation and concentration is identified. Within the micellar region, increasing concentration leads to an increase in the number of *trans* conformations. However, in the hexagonal region, the ratio remains largely constant, indicating little change in conformational behavior. Upon transition to the lamellar phase, an increasing concentration once again translates to an increase in the abundance of *trans* conformations. This overall behavior is consistent with that observed for similar systems.^11,43^

While the hexagonal–lamellar phase boundary identified via this work is consistent with the previous assignment, the location of the micellar–hexagonal boundary appears at a higher concentration when determined using Raman analysis. While POM identified solutions at 20% concentration as micellar and 28% as hexagonal mesophases, the conformational behavior of 28% solutions fits in more with the trend identified for micellar solutions. This could indicate that the hexagonal phase only becomes well established at concentrations of 35% ≤ *c*. It is possible that the process of POM imaging inadvertently applies some shear during the imaging process, while Raman measurements do not, causing a degree of shear-induced alignment at concentrations on and around the phase boundary.

**Figure 6:**
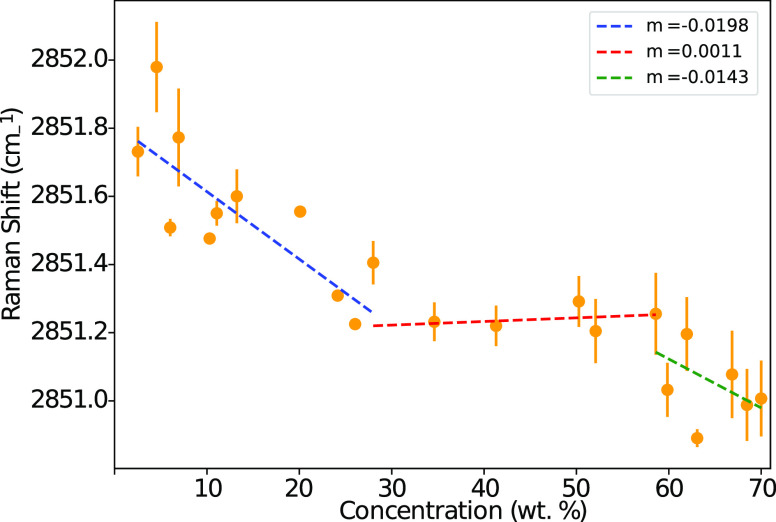
The position of the peak at ≈2850 cm^–1^. Error bars are calculated as the standard error of multiple readings.

The shift from *gauche* to *trans* conformations is also studied by analyzing the position of peak maxima, which can shift due to changes in the magnitudes of overlapping modes. Figure 6 shows the position of the peak located at ≈2850 cm^–1^, which displays a shift with increasing concentration (note that the location of this peak is obtained via a Gaussian fit to the maximum due to the resolution of measurement being only 1 cm^–1^). This shift is thought to result from the ν(CH_2_) symmetric *trans* mode growing with increasing concentration and the influence from the overlapping *gauche* mode at 2876 cm^–1^ becoming less significant. Once again, the location of this peak indicates that there is an increase in *trans* modes with increasing concentration within the micellar and lamellar phases, with minimal change within the hexagonal mesophase. The location of the phase boundaries identified from these peaks is roughly in agreement with those in Figure 5; however, the micellar–hexagonal transition appears to be located at a lower concentration than that using the intensity of the peak. This is in better agreement with the phase assignment from POM imaging in our previous work.^8^

### 3.2 Rheology

**Figure 7:**
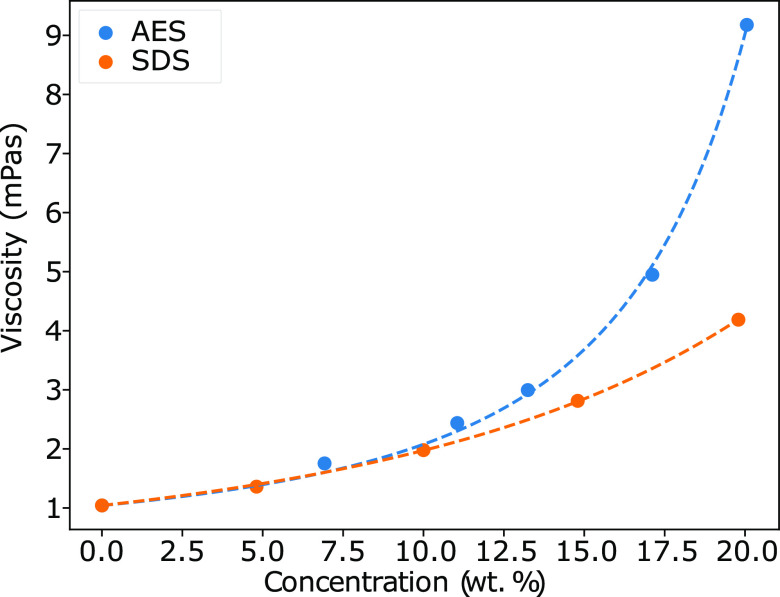
Measured viscosity (mPa·s) against concentration for the AES and SDS solutions. The solutions are fitted using eq 1: *K* = 5.07 and *Q* = 2.65 (AES); *K* = 5.84 and *Q* = 0.86 (SDS).

The micellar solutions are found to exhibit Newtonian behavior in the shear rate range trialled (see the Supporting Information). The relationship between viscosity η and surfactant concentration is shown in Figure 7, where the viscosity for SDS solutions is in agreement with previous authors.^59,60^

The viscosity increases nonlinearly with concentration, for both AES and SDS systems, with the AES solutions possessing a larger viscosity than SDS solutions, in particular, for higher surfactant concentrations. For micellar systems, changes in the solution viscosity are expected to be most influenced by changes in the shape of micellar aggregates, the number of micelles formed, and/or due to micellar interactions. The larger viscosity of AES solutions may be, in part, due to an increase in the micelle size, due to the average length of an AES molecule being approximately one ethoxylation unit (OCH_2_CH_2_) longer.

The relationship is fit to an equation of the form
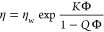
1where η_w_ is water viscosity, Φ is the fraction of surfactant, *Q* is an interparticle parameter, and *K* is a shape factor.^61^ The choice *K* = 2.5 theoretically correlates to what is expected for rigid, spherical micelles,^62^ while deviation from this would indicate prolate or oblate shapes. However, it is theorized that very high values of *K* can also arise from electroviscous effects from the presence of charged micelles.^61^ We note that, if parameter *K* is fixed at *K* = 2.5 and we choose to perform the fit exclusively via *Q*, then we are unable to find a good fit to the data (see the Supporting Information), particularly for AES solutions, indicating nonspherical micelles or increasing intermicellar interactions.

It is of interest that there is a considerable increase in the number of *trans* modes within the micellar region of the phase diagram as the concentration is increased. The significant conformational changes observed for molecules in this region indicate changes in the shape or size of micelles, as opposed to micelles with a constant aggregation number. The increase in *trans* modes within the micellar region is followed by a smooth transition to the hexagonal phase, as opposed to an abrupt one. This also indicates a gradual change in the phase structure, from spherical → nonspherical → worm-like micelles, rather than an abrupt structural change.

### 3.3 Molecular Dynamics

#### 3.3.1 SDS Micelles

In this section, we analyze the results of our simulations of *n* = 0 surfactants in the micellar phase, where our results are obtained both from simulations beginning from random initial configurations and also from those from preformed micelles.

**Figure 8:**
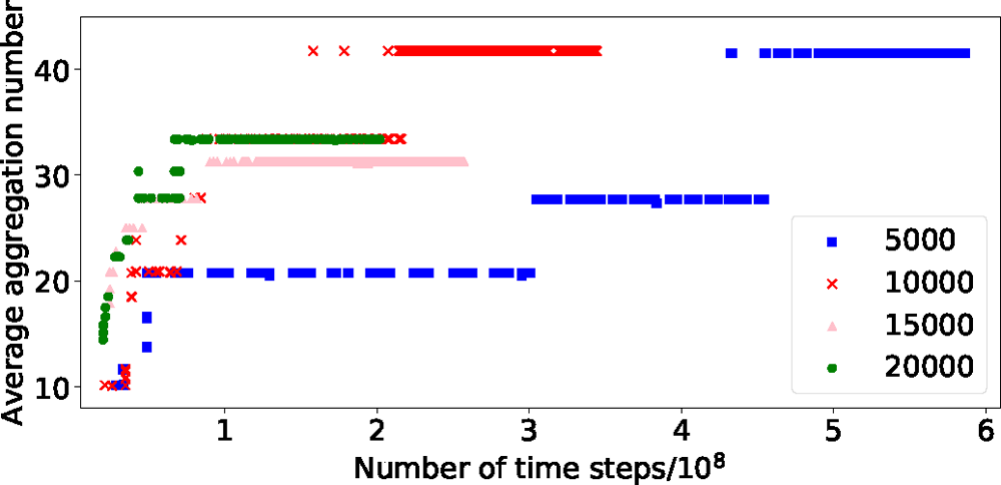
Equilibration of the aggregation number for SDS micelles in boxes of different sizes.

Table 5 provides the aggregation numbers achieved from random initial configurations. For each box size, multiple micelles form in the same simulation box; e.g., in the case where we use 5,000 molecules, there are two coexisting micelles (one with *N* = 28 and another with *N* = 55). Figure 8 shows the equilibration of the aggregation number for various box sizes.

**Table 5:** Micelles Formed from Random Initial Configurations in Simulations of Various Sizes (*c* = 20 wt %)^a^

Size (no. of molecules)	*L* (nm)	Micelles formed (aggregation number)
5,000	5.58	28, 55
10,000	7.03	14, 44, 46, 63
15,000	8.04	14, 22, 22, 28, 28, 43, 43, 50
20,000	8.85	15, 18, 20, 25, 26, 30, 40, 42, 58, 60

a Also shown is the final box edge length *L* after equilibration.

**Figure 9:**
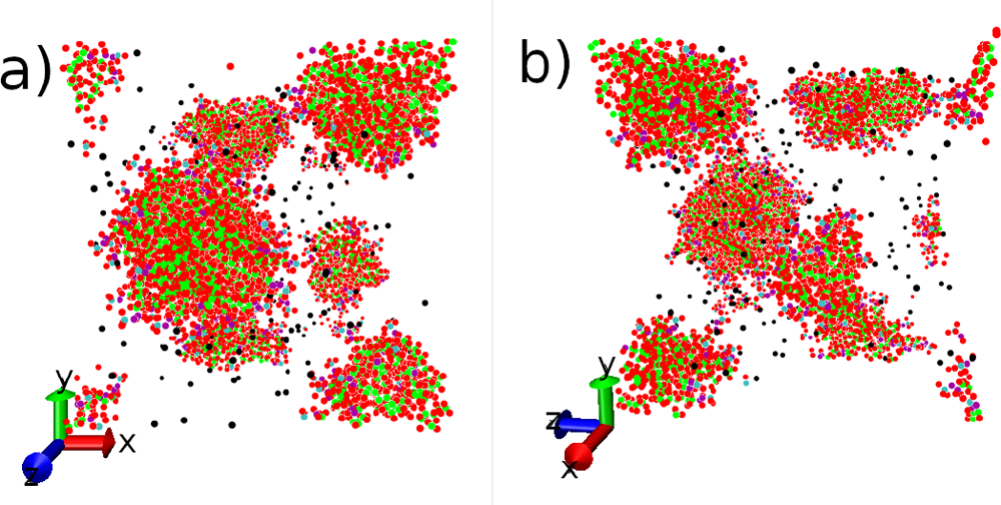
Micelles in a simulation box of size 10,000 from two different orientations. Water molecules are not shown for clarity.

For all box sizes trialled, the mean aggregation numbers achieved are lower than those seen in experiments, where the aggregation number of SDS is reported to be around 100.^63^ This underprediction is to be expected, given the time scales involved in aggregation. Figure 9 shows examples of the observed micelle formation. As the box size increases (and the number of micelles increases), there is a greater number of smaller micelles (*N* ≤ 30) appearing. Two small micelles tend to combine on a shorter time scale than two larger ones do; therefore, one might assume that small micelles would readily combine in all simulation cases, e.g., micelles *N* = 15 and *N* = 18 in the box *N*_T_ = 20,000. However, one of the main barriers to small micelles combining is the physical distance between them. In the example given, the two micelles are located on opposite sides of the domain and would therefore have to diffuse a large distance to combine. This is on a time scale which is larger than we can simulate.

In order to get around the problem of small aggregation numbers, we also performed simulations where we preassemble the micelle at sizes closer to the experimental aggregation number. These simulations equilibrate extremely quickly (compared to the equilibration process for the random initial configurations), where equilibration is determined by monitoring various conformation properties and micellar shapes (see below) as a function of simulation time. The generation of a distribution of micelle sizes means that we can study various properties as a function of aggregation number.

In order to compare directly Raman spectra data, we analyze molecular conformation by calculating the dihedral angles between bonded carbon beads, calculating the ratio of the dihedral angles *trans*/*gauche* for different simulation cases. Theoretically, *trans* and *gauche* conformations can be described using their dihedral angles, defined as follows. For a series of bonds labeled ..., *i* – 1, *i*, *i* + 1, ..., the rotational angle of bond *i* is defined as the angle between two planes. The first plane is defined by bonds *i* – 1 and *i* and the second plane by bonds *i* and *i* + 1. This angle is illustrated in Figure 4. Special cases of this angle θ include θ = 180°, θ = +60°, and θ = −60°, which are the *trans*, *gauche*^+^, and *gauche*^–^ conformations. In our simulations, we define *gauche* angles as those between 30° and 90° or −30° and −90°. *Trans* angles are defined as being between ±150° and 180°. While angles outside of these ranges can occur, they are ignored for the purpose of calculations in this work. A more common approach for studying conformation in simulations across the existing literature is using the end-to-end distance *R* of molecules. We define *R* as the distance between the sulfur atom and the final carbon atom in the tail. We calculate *R*, as well as the *trans*/*gauche* ratio, for comparison between the two.

**Figure 10:**
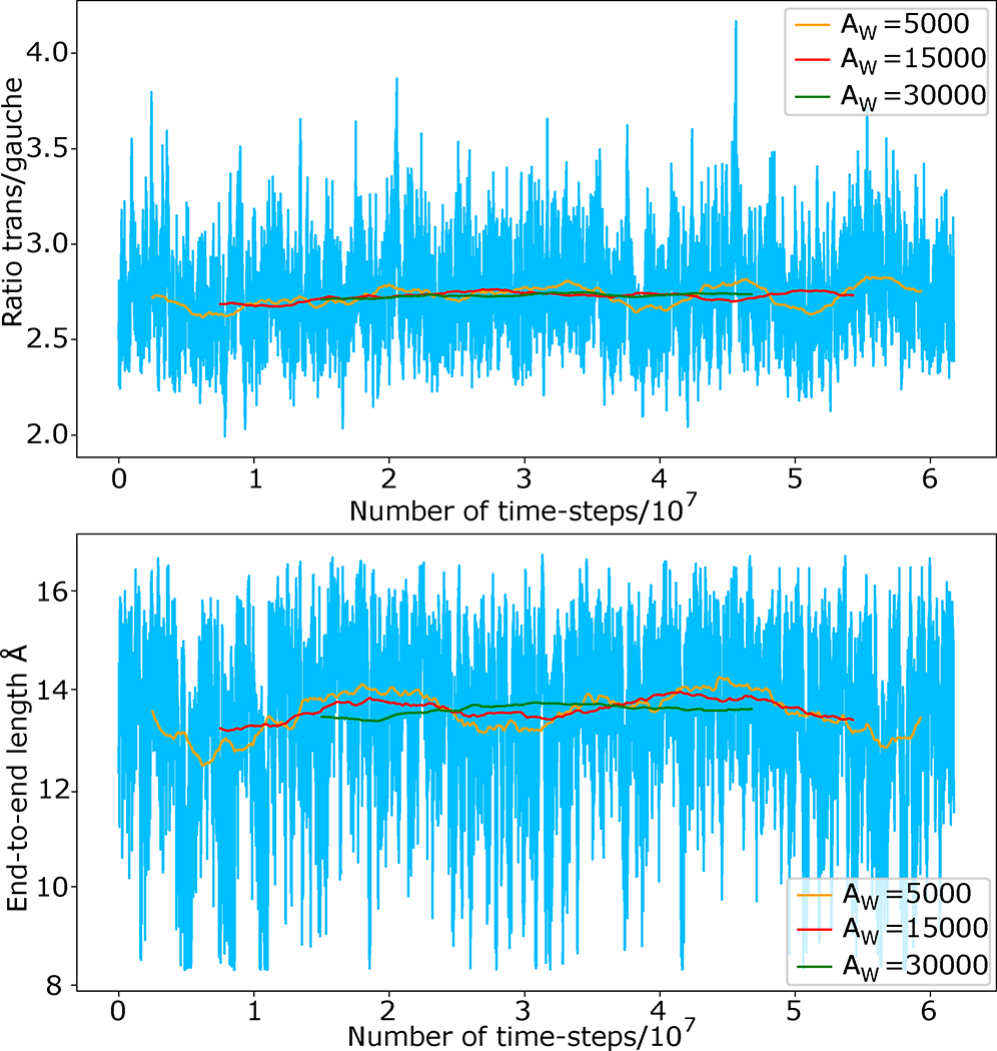
An example of the equilibration of the end-to-end length and *trans*/*gauche* ratio. Example shown for a preassembled micelle of SDS with *N* = 100. We plot the average value (over total surfactant molecules) against the time step. We also overlay a moving average with window *A*_W_.

**Figure 11:**
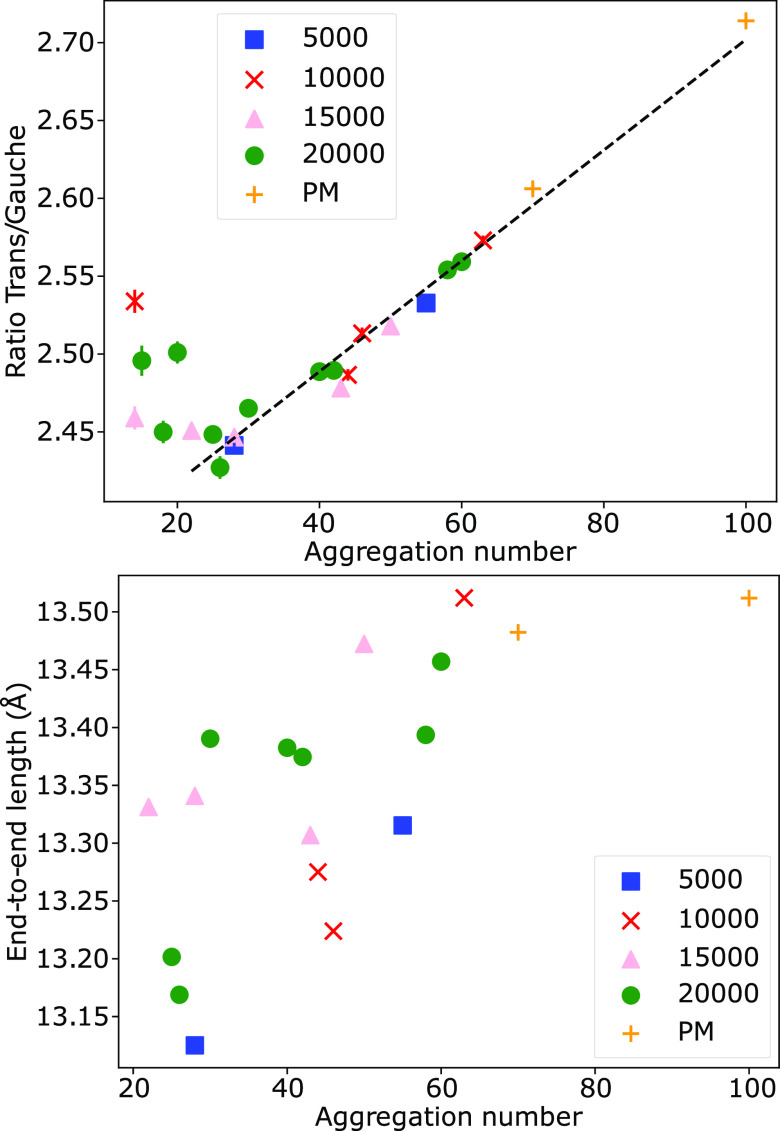
Relationship between the ratio of *trans*/*gauche* conformations with aggregation number and end-to-end length for micelles with *n* = 0 (SDS). The data plotted is from simulations initialized with random initial configurations (5,000, 10,000, 15,000, 20,000) and prearranged micelles (PM).

We monitor the equilibration of the preassembled micelles by monitoring the end-to-end length and the *trans*/*gauche* ratio, which is shown in Figure 10. We find that a fairly large number of outputs are required for calculating precise values. The *trans*/*gauche* ratio is plotted against aggregation number in Figure 11, where we observe an increase in the relative number of *trans* conformations with aggregation number. When the aggregation number is above *N* ≈ 20, the relationship between the *trans*/*gauche* ratio and the aggregation number is determined to be linear. In contrast, the end-to-end length displays a less clear relationship with aggregation number. The results indicate that the end-to-end length approximately increases with aggregation number; however, the magnitude of change is much smaller compared with analyzing the ratio of *trans*/*gauche*. This suggests that dihedral angles are a more reliable way to assess conformational changes.

**Figure 12:**
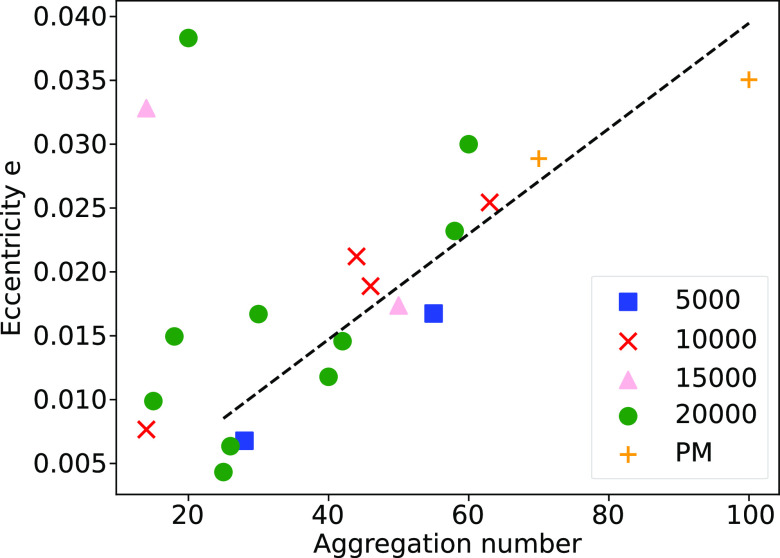
Relationship between the eccentricity *e* and aggregation number for micelles with *n* = 0 (SDS). The data plotted is from simulations initialized with random initial configurations (5,000, 10,000, 15,000, 20,000) and prearranged micelles (PM).

In order to characterize the shape of the micelles, we calculate the moment of inertia *I* in the *x*, *y*, and *z* directions. A spherical micelle is indicated by *I*_*x*_ ≈ *I*_*y*_ ≈ *I*_*z*_. To quantify the sphericality, we also calculate the micelle’s eccentricity *e*, defined as
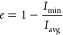
2where *I*_min_ is the smallest moment of inertia calculated in the *x*, *y*, and *z* directions and *I*_avg_ is the average of all three *I*. Spherical micelles will possess a value which is close to zero. The relationship between *e* and aggregation number is shown in Figure 12, showing that the micelles are largely spherical, although they do appear to be becoming more nonspherical with aggregation number, which is in agreement with our previous DPD work.^8^ We expect that the aggregation number needs to become significantly larger, in order to see any significant deviations from nonspherical behavior, which is also in agreement with experimental results^63^ and our previous simulations.^8^

#### 3.3.2 Lamellar Phases, SLE1S Micelles, and Free Monomers

In this section, we look at the conformational behavior of free monomers compared with when surfactants are in micelles. We also look at a small number of simulations of *n* = 1 (SLE1S) surfactants, to compare with the results obtained for *n* = 0 surfactants which are analyzed in the previous section. Finally, here we also look at the conformational behavior of both *n* = 0 and *n* = 1 surfactants in the lamellar phase, compared with the micellar phases.

Equilibration of the lamellar phase should result in a layer of water molecules and a parallel bilayer of surfactant molecules, where the surfactant tails are orientated toward the center of the surfactant layers. However, for the *n* = 0 case, the bilayer does not separate into two distinct layers of surfactant and water molecules. Instead, the water layer is perforated by the surfactant layer, where a bridge exists between the surfactant layers. This is not too surprising, given that experimentally the lamellar phase is unstable and does not form for SDS. However, for the *n* = 1 case, the surfactant layer and water layer are completely separated and perfectly parallel.

Table 6 summarizes the determined ratio and end-to-end lengths for the different cases studied (i.e., micellar phase, lamellar phases, and also free monomer simulations). We observe that there is a significant change in the conformation upon micelle formation. Subsequent changes to conformation with an increasing aggregation number are relatively subtle. However, there is once again a large jump in the ratio of *trans*/*gauche* (and end-to-end length) upon transition to a lamellar phase. This correlates well with our experimental results. In general, while the end-to-end length for the *n* = 1 cases is larger than that for the *n* = 0 cases (due to extension from the additional ethylene oxide group), the number of *trans* conformations in the hydrocarbon tail is lower than that for the shorter molecule.

**Table 6:** Results of the Ratio of *trans*/*gauche* Conformations and End-to-End Length for Molecules of Various Ethoxylation (*n*) for Different Phases

Molecule	Phase	T/G ratio	End-to-end length (Å)
*n* = 0	Monomer	1.93	12.2
	Micellar (random initialization)	2.43–2.57	13.1–13.5
	Micellar (*N* = 70)	2.61	13.4
	Micellar (*N* = 100)	2.71	13.5
	Lamellar	3.45	14.5
*n* = 1	Monomer	1.88	13.5
	Micellar (*N* = 50)	2.51	15.2
	Micellar (*N* = 100)	2.79	16.2
	Lamellar	2.93	16.2

## 4 Conclusions

In this work, we study the effect phase structure has on the conformational behavior of surfactant solutions, where existing literature comparing conformational behavior across the full phase diagram is limited. While many experimental methods are used across the literature to determine the type of phase structure which is present, such as polarized optical microscopy, rheological measurements, small-angle scattering, etc., it is usually difficult to determine the exact location of these boundaries. Often different experimental techniques indicate conflicting results, which can be the result of mixed phases or difficulties in sample preparation for an experimental technique (e.g., the application of shear). This conformational study aims to aid in the understanding of the structure of mesophases and also to help understand the transition between phases.

Using Raman spectroscopy, within the micellar region of the phase diagram we find that the ratio of *trans* to *gauche* conformations increases with concentration and largely plateaus in the hexagonal region. Following a transition to the lamellar phase, the ratio of *trans* to *gauche* conformations increases once again with increasing concentration. It was reported in our previous work^8^ that there is a decrease in hexagonal inter-rod spacing with increasing surfactant concentration. Therefore, the unaltered conformational behavior in the hexagonal region of the phase diagram is due to the fact that the packing of surfactant molecules within individual rods remains virtually unchanged with varying concentration, with just the separation between individual rods being influenced. This is in contrast to both the micellar and lamellar phases, where increasing the concentration is expected to lead to altered packing of surfactant molecules, thus influencing their conformation.

The increase in viscosity for AES micellar phases was indicated (from fits to theoretical equations) to be a result of either shape/size micelle changes or an increase in the number of micelles (with a relatively constant relationship between aggregation number and concentration), leading to more intermicellar interactions. The increase in *trans* modes with the concentration in the micellar region indicates that this is a result of significant shape and size changes for AES micelles. We suggest that these shape changes contribute significantly to the increase in viscosity observed for AES solutions with concentration. This is of interest due to the widely varying and conflicting literature surrounding the nature of micelles formed by SDS (and sodium lauryl ether sulfate) surfactants. Fits to experimental small-angle scattering data can often be challenging, and therefore, a wide variety of results have been obtained for the shape of SDS micelles.^63−67^

One of the drawbacks of using Raman spectroscopy to study conformational behavior is that it is considered nontrivial to assign spectra peaks to molecular conformations, particularly for complex molecules which produce multiple overlapping peaks. In addition, the exact relationship between the magnitude of peaks in the spectra and the relative abundance in solution is generally unknown. It has also been the case that historically there has been disagreement about the origin of particular peaks, complicating experimental analysis. Therefore, we performed a selection of simulations using molecular dynamics to investigate the impact of phase formation on conformation.

Using molecular dynamics, we are able to investigate the impact that micelle formation has on the conformation of the molecule. There is a large increase in the number of *trans* conformations upon micelle formation (compared with that of a free monomer). This is of interest, since it is difficult to investigate the conformational behavior of free monomers using Raman spectroscopy, since at low concentrations the signal-to-noise ratio becomes inhibitively small. We then show that there is an increase in the number of *trans* modes with increasing aggregation number so that the increase in *trans* modes we observe via Raman spectroscopy measurements could be corresponding to an increase in micelle size and aggregation number. This supports our suggestion that the large increase in viscosity (with increasing concentration) is due to a significant change in micelle shape or size.

## Supporting Information Available

Figure S1 - the viscosity against shear rate for AES and SDS solutions in the micellar phase; Figure S2 - the viscosity against concentration for AES and SDS solutions, fitted with different theoretical relationships (PDF)
