# Supplementary material for: Using Raman Spectroscopy and Molecular Dynamics to Study Conformation Changes of Sodium Lauryl Ether Sulfate Molecules
Source: J Phys Chem B. 2023 May 16;127(20):4676–86. doi: 10.1021/acs.jpcb.3c02022 (PMC10226124; doi:10.1021/acs.jpcb.3c02022)
Supplement: Supplementary file 1 — jp3c02022_si_001.pdf [file jp3c02022_si_001.pdf]

Supporting Information

Using Raman Spectroscopy and Molecular Dynamics to Study Conformation Changes of Sodium Lauryl Ether Sulfate Molecules

Authors: Rachel L. Hendrikse* [1 2], Andrew E. Bayly[1], Peter K. Jimack[2] and and Xiaojun Lai[1].
Affiliations: [1] School of Chemical and Process Engineering, University of Leeds, Leeds, United Kingdom, [2] EPSRC Centre for Doctoral Training in Fluid Dynamics at Leeds, University of Leeds, Leeds, United Kingdom

Viscosity measurement of micellar AES solutions

Viscosity measurements in the AES micellar phase were made for concentrations 7, 11, 13, 17, 20 AES wt.%. Measurements were also made for a range of SDS solutions with concentrations 5, 10, 15, 20 wt.%, as well as for pure water. Although the viscosity of SDS micellar solutions has been reported by other authors, they are repeated here to allow for a direct comparison with AES solutions, measured using the same procedure and equipment. Measurements were performed at 25°C using the Anton Paar Physica MCR301 Rheometer and 27mm concentric cylinder geometry.

A logarithmic step-wise ramp method was used in order to gradually increase the shear rate, starting at a shear rate of 0.01s^{-1} (although in every case the shear rate had to reach higher values before a large enough torque value was measured for viscosity calculation). Once the shear rate had reached 100s^{-1} the results were repeated in order to check for thixotropic behaviour, but the results were found to be independent of past shearing. A plot of the calculated viscosity against shear rate is shown in Fig. S1, and it can be seen that in the shear rates trialled, the solutions exhibit Newtonian behaviour.

1 Fitting viscosity as a function of concentration

Various relations are trialled for the relationship between concentration and viscosity, for SDS and AES solutions, shown in Fig. S2. An equation of the form $\eta = \eta_\text{w}(1+2.5\Phi+6.2\Phi^2)$ is shown to be a poor fit to the data. This is to be expected, as this relation is not expected to be valid beyond very low concentrations. The Mooney equation ($\eta = \eta_\text{w} \exp \frac{2.5\Phi}{1-Q\Phi}$) provides a more reasonable fit to the data, however a much better fit is obtained when K is allowed to deviate from 2.5.

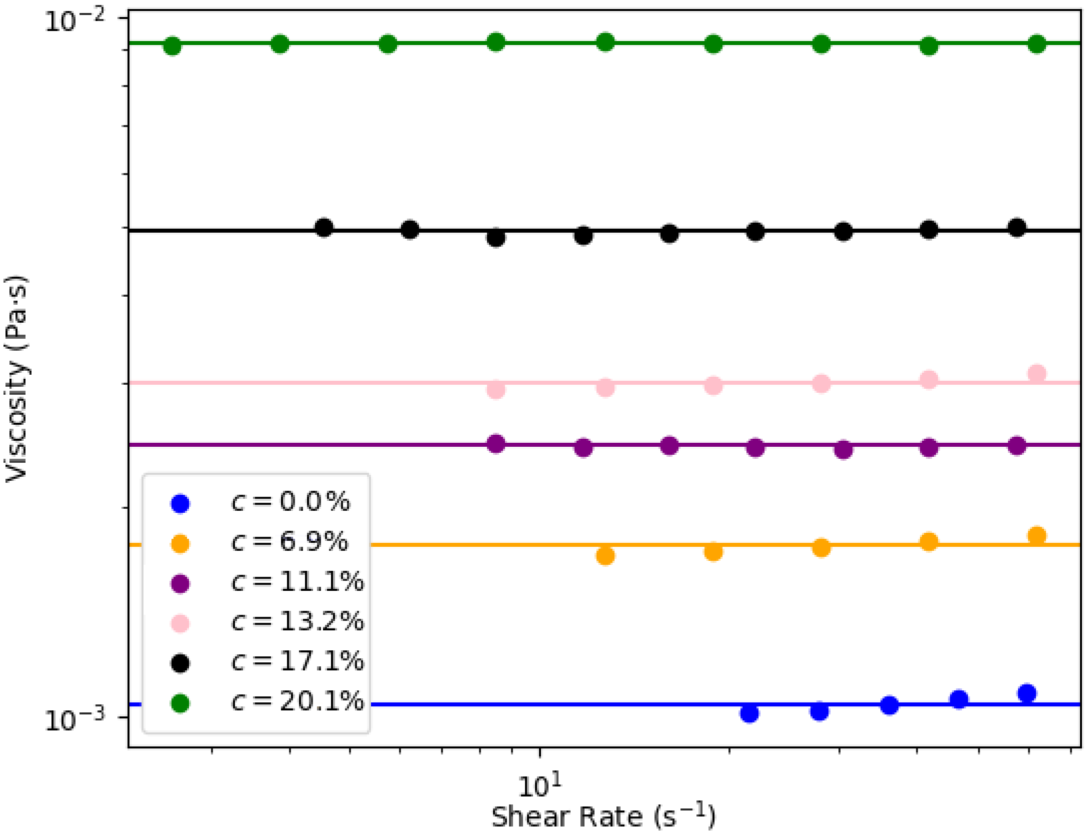

(a) AES solutions

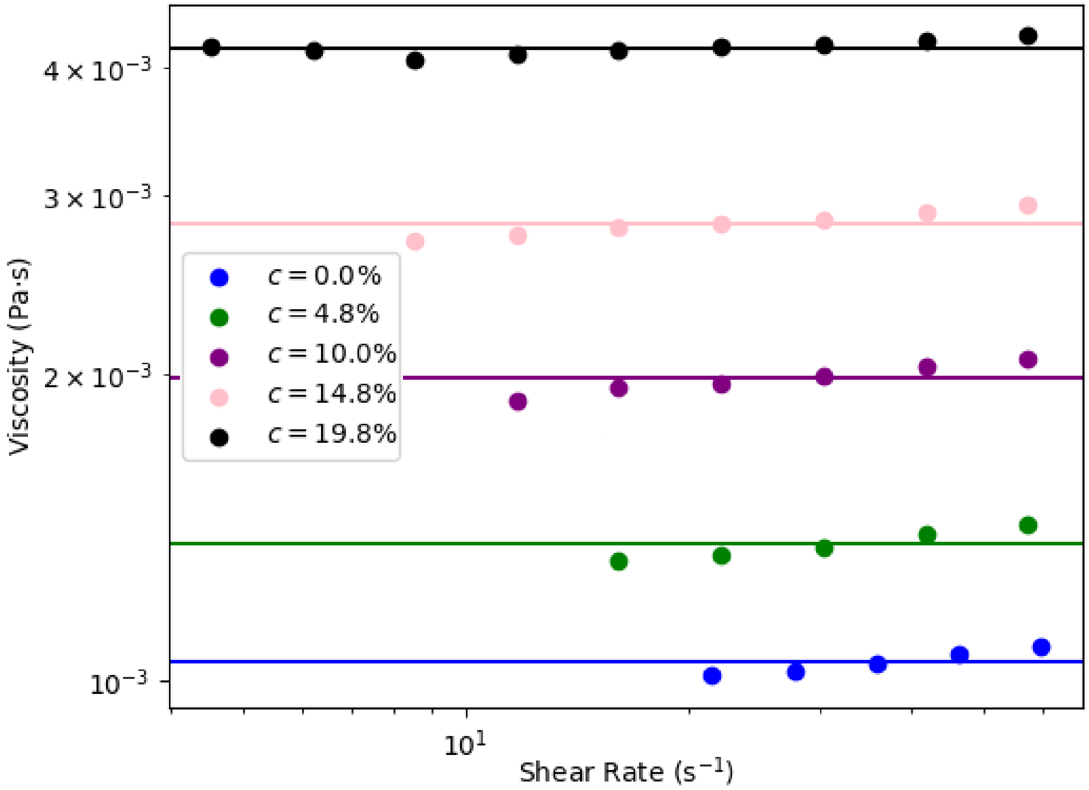

(b) SDS solutions

Fig. S1: Calculated viscosity against shear rate for a range of (a) AES and (b) SDS solutions in the micellar phase, for a variety of concentrations c. Solutions display Newtonian behaviour.

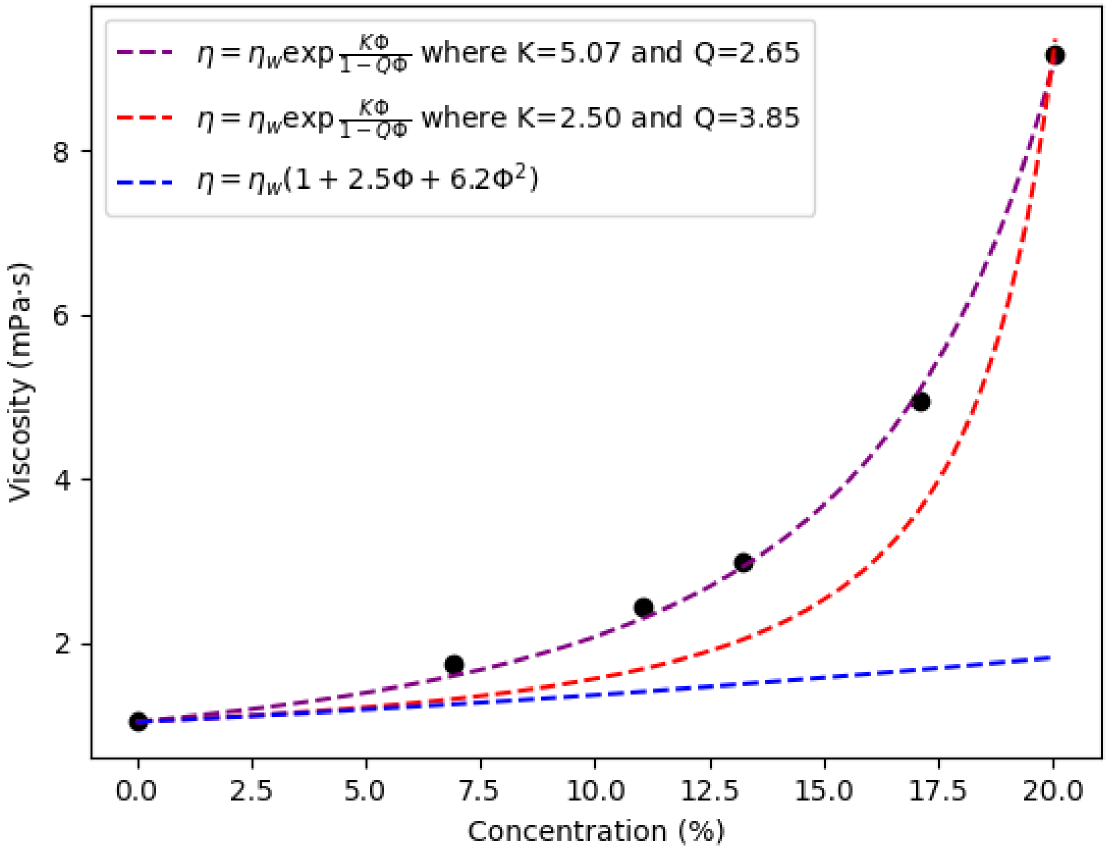

(a) AES solutions

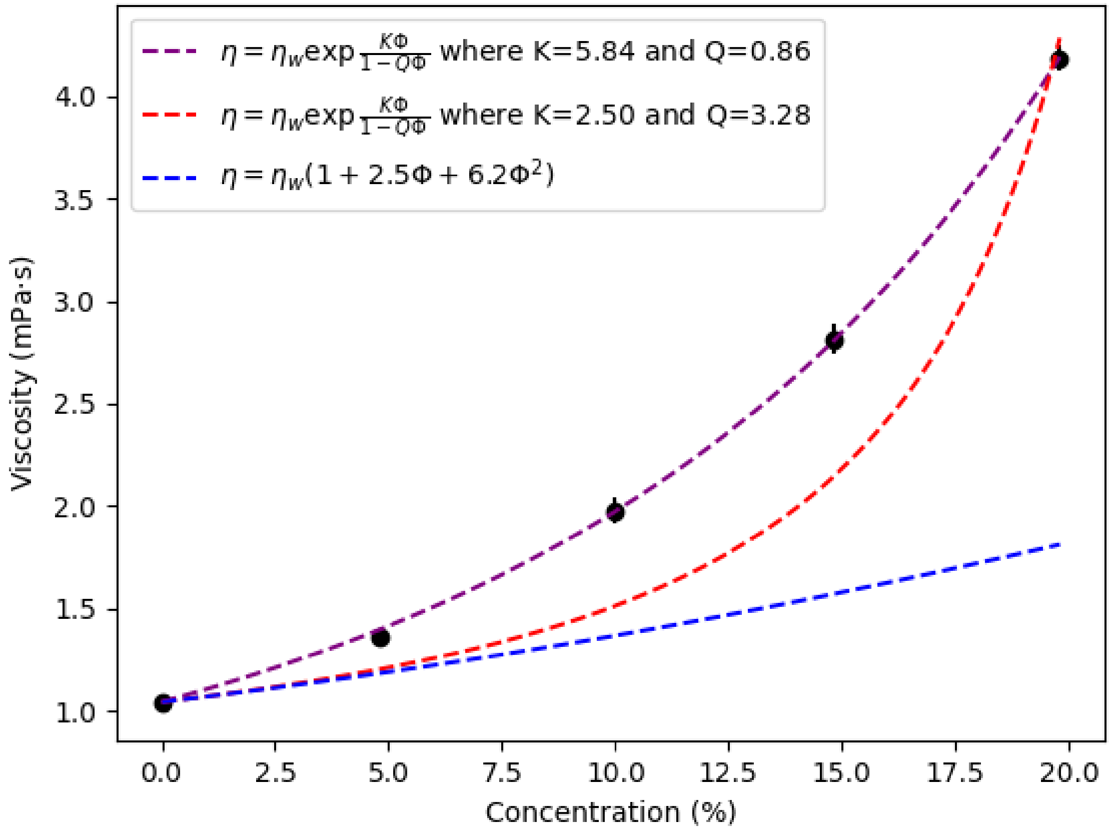

(b) SDS solutions

Fig. S2: Plots of calculated viscosity (mPa·s) against concentration (wt.%) for AES and SDS solutions: (a) AES and (b) SDS solutions fitted with various relations. Error bars are calculated as the standard deviation of the results obtained from varying shear rates (Fig. S1), but are smaller than the symbol size so are not visible.
